# CHMP6 as a novel prognostic biomarker in bladder cancer: insights from a comprehensive cell death-related gene risk model

**DOI:** 10.3389/fonc.2025.1564826

**Published:** 2025-06-24

**Authors:** Wei Ning, Yan-Ru Yang, Chen-Xu Wu, Yue Hou, Zhi-Wei Liu, Chang-Bin Yang

**Affiliations:** ^1^ Medical Innovation Center, Air Force Medical University, Xi’an, China; ^2^ Department of Pathology, the First Affiliated Hospital of Air Force Medical University, Xi’an, China; ^3^ School of Life Information Science and Technology, ShanghaiTech University, Shanghai, China

**Keywords:** CHMP6, BLCA, CDRI, ICD, ferroptosis

## Abstract

**Introduction:**

Bladder cancer (BLCA) is a prevalent and aggressive disease characterized by substantial molecular heterogeneity, complicating its diagnosis and treatment. Existing therapies, including surgery and chemotherapy, often lack specificity. Alterations in cell death mechanisms, such as ferroptosis, cuproptosis, and immunogenic cell death, significantly impact cancer progression and prognosis.

**Methods:**

We analyzed gene expression data from TCGA and GEO. Cox regression analyses generated a prognostic risk score model incorporating LIPT1, ACSL5, and CHMP6. This model successfully stratified BLCA patients into different risk categories and was validated through survival analysis, immune infiltration, mutation burden assessment, drug sensitivity predictions, and single-cell analysis. The high-risk group was linked to differentiation processes, developmental stages, and active metabolic pathways.

**Results:**

Experimental validation highlighted CHMP6’s role in enhancing BLCA cell survival and migration by regulating the cell cycle. The model’s prognostic relevance was further supported by drug sensitivity and immune metrics. These results provide valuable insights into potential biomarkers and therapeutic targets for BLCA treatment.

**Discussion:**

The CHMP6 protein promotes BLCA cell survival and invasive migration through modulation of the cell cycle.

## 1 Introduction

Bladder cancer is the second most common malignant tumor of the urinary system globally, with an annual incidence rate exceeding 540,000 cases and a mortality rate of approximately 200,000 deaths per year. It ranks as the ninth most prevalent malignant tumor and the thirteenth leading cause of cancer-related deaths worldwide (1, 2). Currently, the standard treatment for bladder cancer involves surgical resection followed by chemotherapy (3). However, the selection of chemotherapy agents remains largely at the discretion of the treating physician (4). Furthermore, the anticipated length of survival has a significant influence on the selection of active or conservative treatment options for patients (5). Currently, there are numerous prognostic categories for bladder cancer, including the Uromol-2016 classification (6), the Van-Kessel classification (7), the Seiler classification (8), and others. However, the substantial heterogeneity of bladder cancer poses a significant challenge in molecular classification. Consequently, an enhanced insight into the regulatory mechanisms driving the onset and progression of bladder cancer, the identification of molecular markers for prognostication, and the discovery of novel biological targets for targeted therapy are crucial for advancing the prevention and treatment of bladder cancer.

Molecular alterations that affect the mechanisms of cell death are frequently observed in the development of cancer. These alterations permit malignant cells to evade the effects of intrinsic death signals (9). Nevertheless, an increasing body of evidence suggests that there are multiple alternative mechanisms that coordinate various death pathways. Ferroptosis is an iron-dependent form of programmed cell death, marked by uncontrolled peroxidation of phospholipids. The occurrence of this process is primarily contingent upon the elevation of phospholipid-containing polyunsaturated fatty acid chains (pufa-pl), metabolite reactive oxygen species (ROS), and iron accumulation (10). Several studies have shown a relationship between ferroptosis-related genes and cancer prognosis (11, 12). Additionally, Cuproptosis is a unique form of programmed cell death that distinguishes itself from other well-defined cell death processes, the regulatory process is directly connected to mitochondrial metabolism (13). Several studies have established a strong association between copper death-related genes and the onset and progression of cancer (14, 15). Immunogenic cell death (ICD) is a type of tumor cell death induced by the stress caused by certain chemotherapeutics, radiotherapy and oncolytic viruses (16). Many literatures have confirmed that immunogenic death-related genes have great prognostic value (17, 18). While ferroptosis, cuproptosis, and ICD have been extensively studied as independent cell death modalities, their interplay in the prognosis of bladder cancer remains unexplored.

This study holds a novel research perspective on comprehensive cell death mechanisms, with the objective of identifying new molecular markers for bladder cancer prognosis based on a comprehensive cell death model, utilising readily accessible resources in the clinical setting. Additionally, the study aims to elucidate the mechanism through which comprehensive cell death affects the development of bladder cancer. By examining clinical tumour samples, this project would elucidate aspects such as tumour heterogeneity, mutation map, drug sensitivity, and other factors related to comprehensive cell death mechanisms. This would provide a novel perspective on the efficacy of comprehensive death-related oncogene targeted therapy and the advancement of novel therapeutic approaches.

## 2 Materials and methods

### 2.1 Bulk RNA-seq analysis

#### 2.1.1 Dataset collection

Gene expression profiles and clinical data of bladder cancer patients were sourced from the Cancer Genome Atlas (TCGA) database (https://portal.gdc.cancer.gov/). We also consulted to obtain information on ferroptosis, cuproptosis, and immunogenic death-related genes (**Supplementary Table S1**). Furthermore, gene expression profiles and clinical data of the GSE31684, GSE32548 and GSE32894 datasets were acquired from the Gene Expression Omnibus (GEO) database (https://www.ncbi.nlm.nih.gov/geo/).

#### 2.1.2 Prognostic risk characteristics construction

Firstly, the “edger” (19) was employed to analyse the discrepancy between BLCA samples and normal samples. Genes with a log2 fold change (log2fc) greater than 1 and a false discovery rate (FDR) below 0.05 were regarded as differentially expressed. Subsequently, the intersection analysis with comprehensive death-related genes was conducted to ascertain the differential comprehensive death-related genes. Then the univariate Cox regression analysis was employed to identify comprehensive death-related genes associated with overall survival (OS) in patients with BLCA. To identify comprehensive death-related genes that independently influence the prognosis of BLCA, We conducted a multivariate Cox regression on the selected genes. The correlation coefficient between each gene was calculated. A comprehensive mortality prognostic risk score for BLCA was constructed, and a formula was established as follows:


$$\begin{array}{c} RiskScore=coef(LIPT1)*\exp(LIPT1)\\ +coef(ACSL5)*exp(ACSL5)\\ +coef(CHMP6)*\exp(CHMP6) \end{array}$$

#### 2.1.3 Survival analysis

A survival analysis was conducted on various subgroups of BLCA and TCGA comprehensive death risk scores for other cancer types. The R packages “survival” and “survminer” (20) were employed to generate Kaplan-Meier curves.

#### 2.1.4 Enrichment analysis of high and low risk groups

The R packages “edgeR” and “ggplot2” (21) were utilized for the analysis and visualization of differentially expressed protein-coding genes across the distinct risk groups. To further explore the potential biological functions, We performed GSEA, GO enrichment, and KEGG pathway analysis. The R packages “msigdbr” (22), “fgsea” (23), “clusterProfiler” (24) were used to generate the enrichment results.

#### 2.1.5 Analysis of immune infiltration

The tumour tissue transcriptome data underwent quantitative transformation via “CIBERSORT” (25), xCell (26) and TIMER (27) analysis, allowing the assessment of human immune cell subsets. Immune cell profiles were compared between high-risk and low-risk groups to assess tumor microenvironment differences. The Wilcoxon test was applied to identify significant variations in immune cell infiltration and associated functional characteristics between the two groups.

#### 2.1.6 Somatic variant analysis

Somatic mutation data, derived from whole exome sequencing of the TCGA-BLCA dataset. The mutation annotation format (MAF) files, containing information on single nucleotide variants, were analyzed using the “maftools” R package (28).

#### 2.1.7 Pharmacological response assessment

The “pRRophetic” package (29) was employed to evaluate the response of each sample to a range of pharmacological agents. The Wilcoxon test was used to assess drug sensitivity differences between the two groups.

### 2.2 Single cell data analysis

Three single cell datasets, GSE135337, GSE129845 and GSE277524, were collected from GEO database. Single-cell data were analyzed using the “Seurat” R package (30). The expression matrix was filtered using the criteria: ncount_rna > 200, nfeature_rna< 5000, and percent_MT< 5 to exclude overexpressed and low-quality cells. Principal component analysis (PCA) and UMAP dimensionality reduction were performed to cluster the cells, followed by annotation using cluster-specific marker genes. Cell trajectory analysis by Monocle2 (31) package.

### 2.3 Experimental validation protocols

#### 2.3.1 Clinical samples

Primary BLCA tissue samples were obtained from 54 patients who underwent surgery at Xijing Hospital, Air Force Military Medical University (Xi’an, China) between 2019 and 2024. All clinical samples adhered to the Clinical International Staging Guidelines and the Declaration of Helsinki. Informed consent was obtained from all participants. Clinicopathologic information was collected from surgical records and pathology reports. The local ethics committee approved all operations done in this research (ethics approval number: K202201-04).

#### 2.3.2 Cell line cultivation and transfection

The human bladder cancer cell lines 5637 and T24 were obtained from Procell Life Science and Technology (Wuhan, China). 5637 cells were cultured in RPMI 1640 medium (PM150110) supplemented with 10% fetal bovine serum (164210-500), 100 units/mL of penicillin, and 100 units/mL of streptomycin sulfate, and incubated at 37°C in a humidified incubator with 5% CO2. T24 cells were maintained in MEM medium (PM150410) supplemented with 10% fetal bovine serum (164210-500), 100 units/mL of penicillin, and 100 units/mL of streptomycin sulfate, and cultured under standard conditions. CHMP6 knockdown (KD) and control (NC) lentiviruses were obtained from GeneChem (Shanghai, China). Transfection was performed following the manufacturer’s instructions, and transfection efficiency was verified through Western blotting and RT-PCR analysis.

#### 2.3.3 Immunohistochemistry and H-score

Surgical specimens were fixed in paraformaldehyde and embedded in paraffin. The blocks were sliced into 4-μm sections and mounted on slides. Slides were baked at 37°C overnight, deparaffinized in xylene, rehydrated in alcohol, acid repaired, and treated with 3% hydrogen peroxide to block peroxidase activity. The sections were incubated with anti-CHMP6 antibody (Catalog No: 31838-2, SAB, USA) at 4°C for ≥12 hours after being blocked with phosphate-buffered saline (PBS) containing 5% bovine serum albumin for 30 minutes. Protein-antibody complexes were detected and developed using standard rapid EnVision technology (Dako, Denmark). The tissue sections were counterstained with hematoxylin, mounted, and observed under a microscope for imaging.

Immunoreactivity was graded as follows: 0 = absence of staining, 1+ = weak cytoplasmic staining, and 2+ = intense cytoplasmic staining. Two experienced researchers independently evaluated the immunohistochemically stained sections. Samples were classified into high expression (2+) and low expression (1+ and 0) groups according to the staining intensity. H scores were calculated using the formula: H score = 1 × (percentage of 1+ stained cells) + 2 × (percentage of 2+ stained cells) + 3 × (percentage of 3+ stained cells), with a range from 0 to 300.

#### 2.3.4 Western blotting assay

Cells were harvested by scraping or grinding, and proteins were extracted for analysis by Western blotting. Anti-CHMP6 antibodies (31838-2, SAB, Nanjing, China; PA5-145901, Invitrogen, USA), anti-LIPT1 antibody (PA5-57064, Invitrogen, USA), and anti-β-tubulin antibody (1:2000, CST, USA) were used to detect the target proteins. The original image is provided in the **Supplementary Information**.

#### 2.3.5 RT-PCR

RNAiso Plus (TAKARA, Shiga-ken, Japan) was used to extract total RNA, which was subsequently analyzed by reverse transcription-PCR with the SYBR Green II kit (TAKARA, Shiga-ken, Japan). The expression levels of target genes’ mRNA were normalized to the ACTN gene. Thermal cycling conditions included 45 cycles: 15 s at 95°C, 5 s at 95°C, and 30 s at 60°C.

#### 2.3.6 Cell viability assay

BLCA cell proliferation was assessed *in vitro* using the Cell Counting Kit-8 (YEASEN, China). Cells were seeded in 96-well plates at a density of 2 × 10³ cells per well, with a final volume of 100 μL. The plates were incubated for a period of time at 37°C with 5% CO_2_. Next, 10 μL of CCK-8 solution was added to each well, and after 1–4 hours of incubation, absorbance at 450 nm was measured using a microplate reader.

#### 2.3.7 Cell permeabilization assay

Transwell chambers (8-μm pores, Corning, Lowell, MA, USA) were positioned on 24-well culture plates (REF3524, Corning, Lowell, MA, USA). Matrigel (356234, BD, USA) was mixed with serum-free medium and added to the chambers. A 200 μL serum-free cell suspension (3.5 × 10^4^cells) was added to the upper chamber, and 500 μL of cell suspension to the lower chamber. After 24 hours, cells that had invaded into the lower chamber were fixed with anhydrous ethanol, stained with crystal violet, and air-dried. Images were captured in three random fields using an inverted microscope (200× magnification), and the number of cells was counted.

#### 2.3.8 Scratch wound test

5637 cells were cultured to 80%-90% confluence, and a straight-line “scratch” was created using a 200-μL pipette tip to form a cell-free area. The cells were then incubated in serum-free medium for 24–48 hours, and cell migration was observed under a microscope.

#### 2.3.9 Annexin V/PI staining

5637 cells were digested with EDTA-free trypsin and then centrifuged at 500 × g for 5 minutes to terminate digestion. The supernatant was discarded, and the cells were washed twice with PBS. For every 1 × 10_5_ cells, they were resuspended in 500 μL of cell staining buffer (typically PBS containing 1%-3% BSA or FBS). Next, 2-5 μL of PI/Annexin V-FITC staining solution (final PI concentration of 0.5 mg/mL) was added to each sample. The cells were incubated for 10-15 minutes, washed twice with PBS, and then analyzed by flow cytometry. The original image is provided in the **Supplementary Information**.

## 3 Results

### 3.1 Defining multiple cell death genes associated with BLCA prognosis

**Figure 1:**
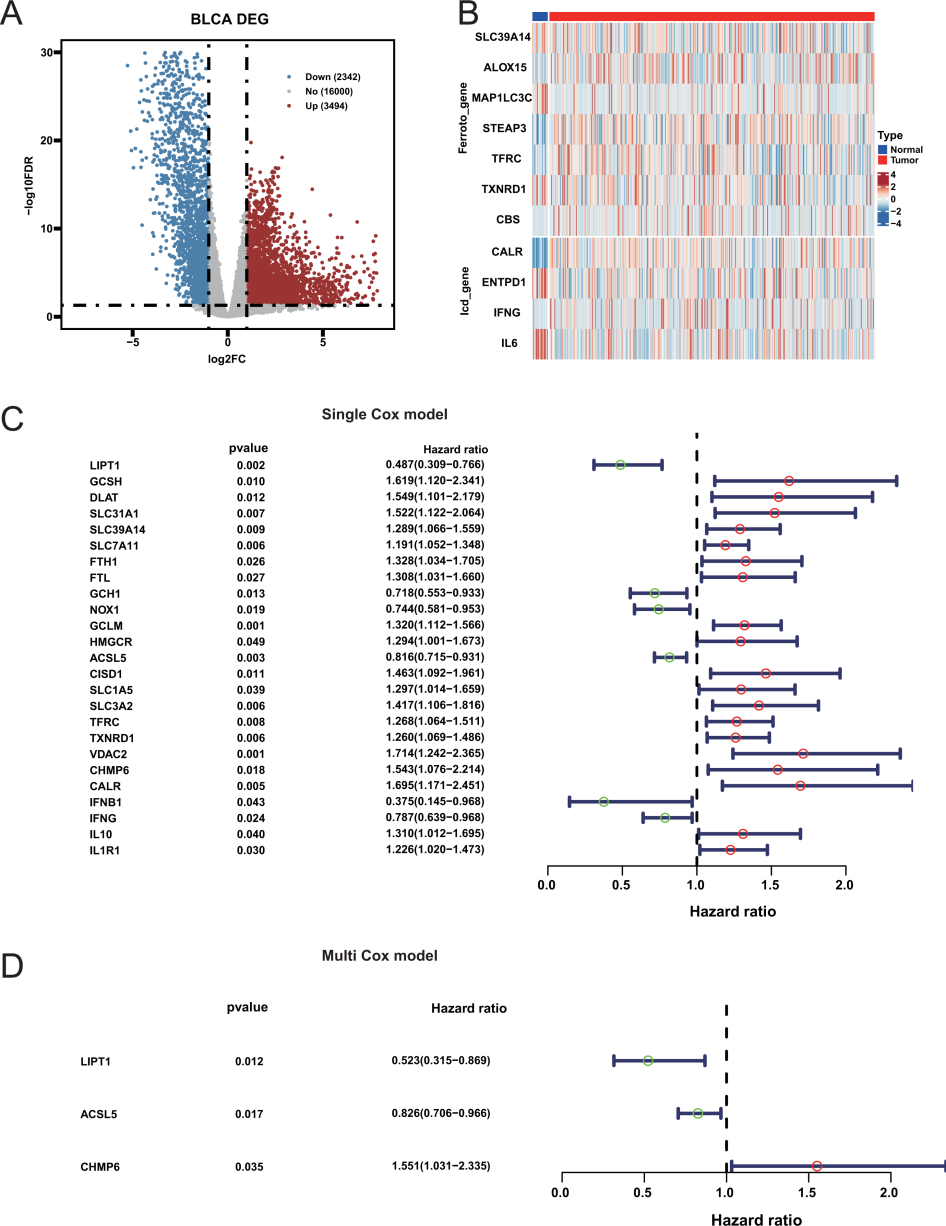
Differential expression and prognostic significance of genes in bladder cancer. **(A)** Volcano Plot of Differentially Expressed Genes between bladder cancer and normal samples. **(B)** Heatmap of Ferroptosis and Immunogenic Cell Death-Related Genes. **(C)** Forest Plot of Univariate Cox Analysis: Hazard ratios and 95% confidence intervals for genes significantly associated with bladder cancer prognosis from univariate Cox analysis. **(D)** Forest Plot of Multivariate Cox Analysis.

The initial analysis of the TCGA database revealed significant differences between BLCA cancer samples and corresponding paracancerous samples (**Figure 1A**; **Supplementary Table S2**). By intersecting with the three cell death related gene sets, it was observed that the three cell death related genes showed no significant difference between cancer tissues and adjacent tissues (**Figure 1B**; **Supplementary Figure S1A–C**), indicating that the cell death activity would not be affected by cancer. Univariate Cox survival analysis was performed on three cell death-related genes to identify those associated with the prognosis of BLCA patients. This analysis revealed that a total of 25 cell death-related genes had a significant impact on the prognosis (**Figure 1C**; **Supplementary Table S3**). Multivariate Cox analysis of these prognostic genes identified three genes that were significantly associated with prognosis. The overall p-value for the likelihood ratio test of the model is 2e-11. Among them, chmp6 is a risk factor, while lipt1 and ACSL5 are protective factors (**Figure 1D**). However, no marked differences in the expression of these three genes were observed between cancerous and adjacent tissues (**Supplementary Figure S1D-F**), suggesting that the prognosis of our patients may not be solely driven by cancer-related factors.

### 3.2 Evaluating the effect of CDRI model on prognosis

**Figure 2:**
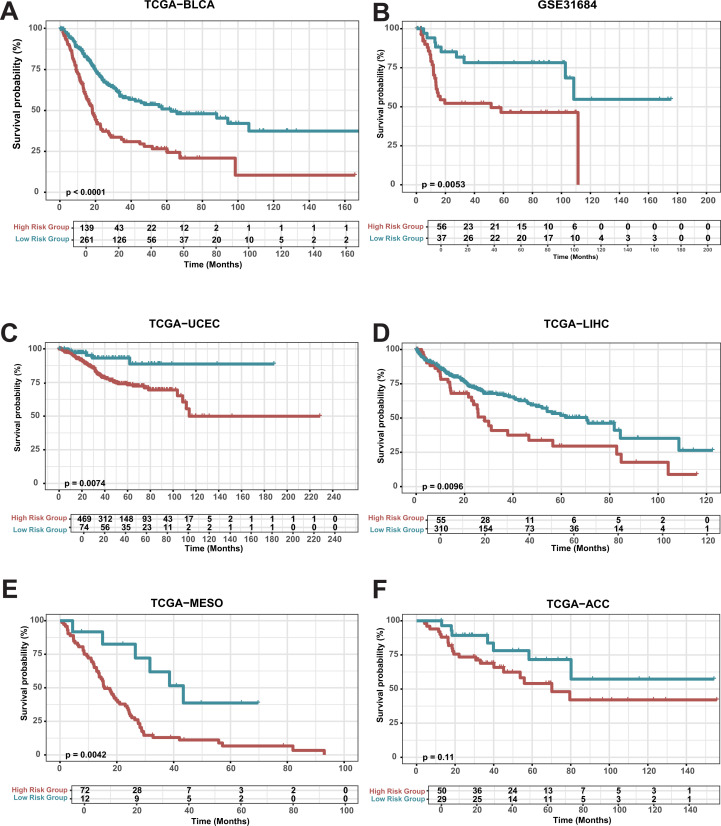
Survival analysis of risk groups **(A)** Overall Survival in TCGA-BLCA: Kaplan-Meier survival curves for high-risk and low-risk groups in bladder cancer (TCGA-BLCA). **(B-E)** Kaplan-Meier survival curves for GSE31684, TCGA-LIHC, TCGA-MESO, TCGA-UCEC, and TCGA-ACC.

A comprehensive death gene risk model (CDRI) was constructed using the genes CHMP6, LIPT1, and ACSL5. Subsequently, cancer patients were divided into high-risk and low-risk groups using CDRI scores (-3.003, 2.369) with a threshold of 0.522 (**Supplementary Table S4**; **Supplementary Table S5**). The prognosis of the high-risk group was markedly inferior to that of the low-risk group (**Figure 2A**). The same trend was observed in additional BLCA datasets upon validation (**Figure 2B**; **Supplementary Figure S2**). To investigate the potential of the CDRI model for pan-cancer prognosis prediction, we extended the model to encompass additional cancer types. Our analysis demonstrated that the prognosis of the high-risk group in TCGA-UECE, TCGA-LIHC, TCGA-MESO and TCGA-ACC was considerably poorer than the low-risk group (**Figures 2C–F**). Previous literature also confirmed that knockout of chmp6 gene would make pancreatic cancer cells and liver cancer cells more sensitive to ferroptosis (32). Our results indicate that the area under the curve for predicting the 1-year, 3-year, and 5-year survival rates of BLAD patients is all above 0.7 (**Supplementary Figure S3A**; **Supplementary Table S6**). Similar results were observed in the validation cohort (**Supplementary Figure S3B–F**). These results indicate that the CDRI model could potentially act as a prognostic biomarker for pan-cancer.

In addition, we compared the CDRI model with Peng’s model (33) and Bo’s model (34), and found that the AUC of the CDRI model was better than that of the Peng’s model at one, three, and five years. Although the AUC of the CDRI model was comparable to that of the Bo’s model on the TCGA dataset, the performance of the CDRI model was much better than that of the Bo’s model on the GSE32548 and GSE32894 datasets. In addition, the Bo’s model uses seven gene features, while the CDRI model only uses three gene features. These results indicate that the CDRI model could potentially act as a prognostic biomarker for pan-cancer.

### 3.3 Research on the mechanism of CDRI model

**Figure 3:**
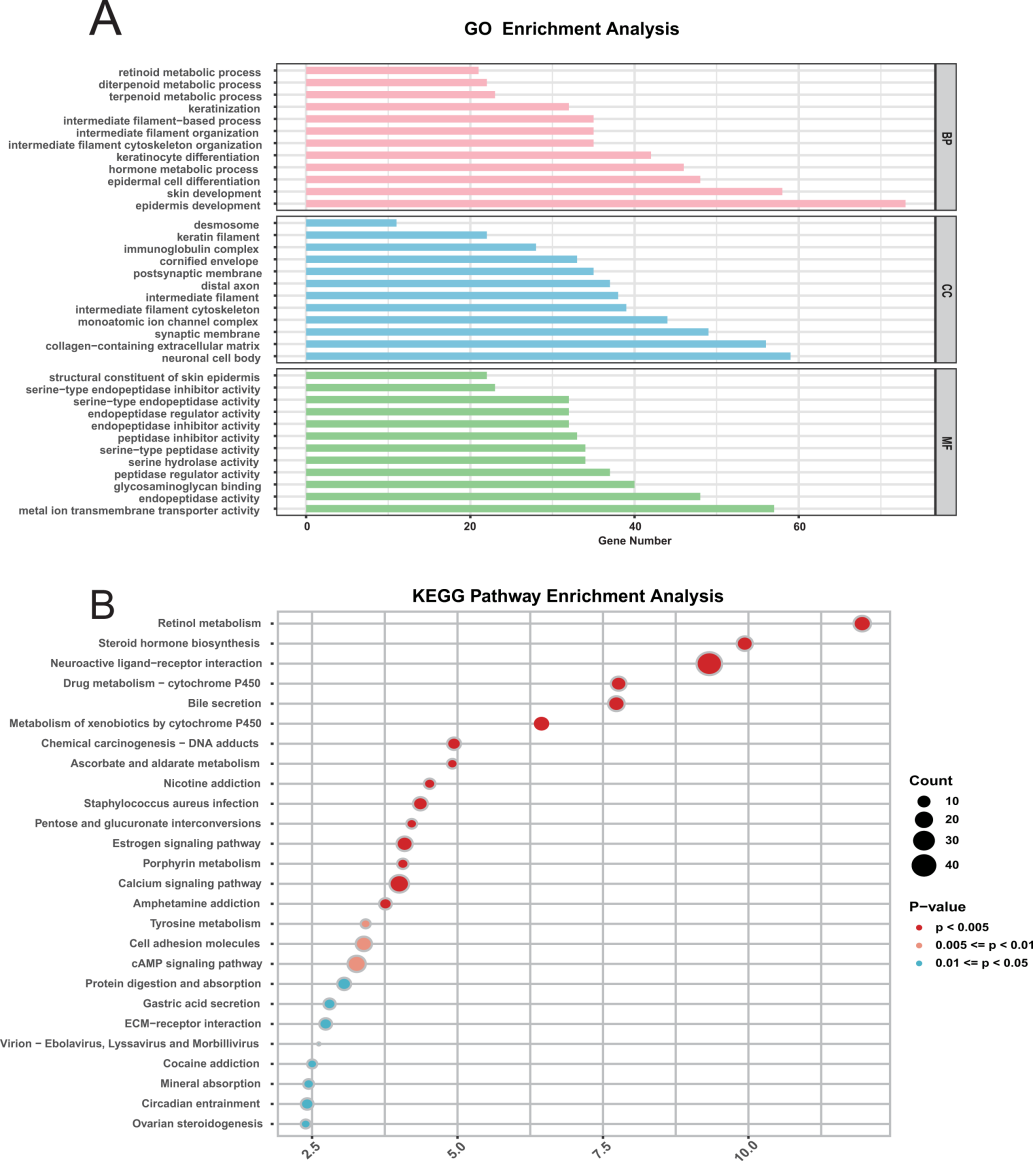
Functional enrichment analysis of differentially expressed genes in bladder cancer. **(A)** GO Pathway Enrichment Analysis: Enriched Gene Ontology (GO) terms for differentially expressed genes include biological processes (BP), cellular components (CC), and molecular functions (MF). **(B)** KEGG Pathway Enrichment Analysis.

To further explore the mechanism behind the CDRI model, we first carried out a comparative assessment between the two groups. Subsequently, our analysis showed that the differences between two groups were predominantly associated with biological processes related to differentiation and development, as well as molecular functions linked to serine peptidase activity (**Figure 3A**). Earlier research has demonstrated that serine peptidase is involved in regulating biological processes related to tumor development (35). KEGG pathway enrichment analysis further indicated that the differences between two groups were primarily enriched in metabolic pathways, including the cAMP signaling pathway (**Figure 3B**). This pathway is also involved in cancer proliferation and progression (36).

### 3.4 Comparison of the tumor immune microenvironment between two groups

**Figure 4:**
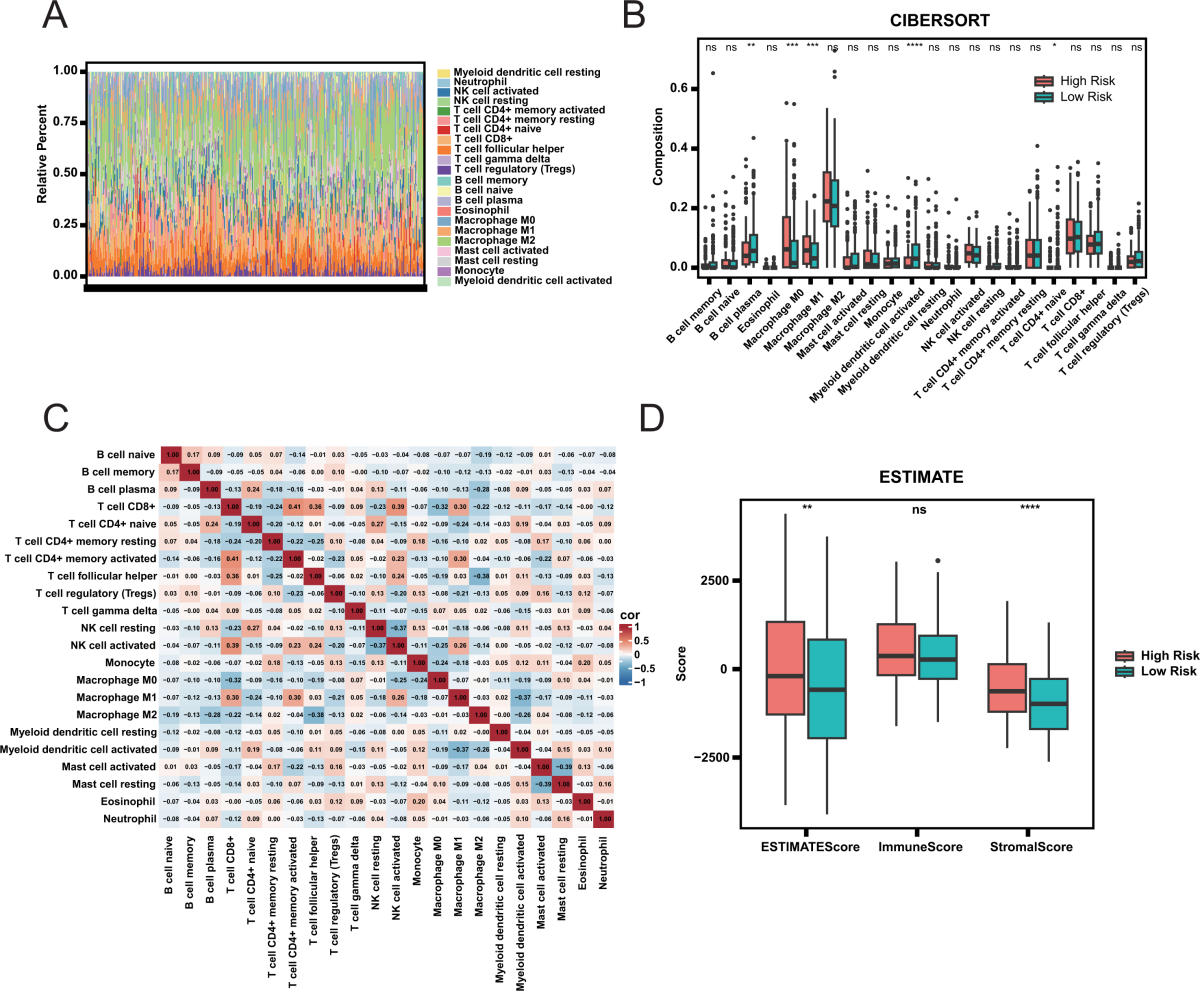
Immune infiltration and tumor microenvironment analysis. **(A)** Relative Abundance of Immune Cell Types in High and Low Risk Groups. **(B)** Comparison of Immune Cell Composition Between High and Low Risk Groups. Statistical significance is indicated by ns (not significant), * (adjust.p< 0.05), ** (adjust.p< 0.01), *** (adjust.p< 0.001), and **** (adjust.p< 0.0001). **(C)** Correlation Heatmap of Immune Cell Types. **(D)** ESTIMATE Scores Comparison Between High and Low Risk Groups.

To determine whether discrepancies exist in the tumor immune landscape between two groups, we estimated the proportions of 22 immune cell types (**Figure 4A**). No significant correlation was observed among the 22 immune cell types (**Figure 4C**), indicating that there was no mixing between cell types. Additionally, there was no significant difference in the proportion of most immune cells between the two groups (**Figure 4B**; **Supplementary Figure S4**), indicating that the difference in prognosis between the two groups was not caused by the proportion of immune cells. The estimated score further indicated no discernible difference in immune scores between the two groups (**Figure 4D**). However, the stromal score in the high-risk group was significantly higher than that in the low-risk group, indicating greater tumor purity in the high-risk group. We speculate that the prognosis of patients may be mainly affected by the purity of tumor, rather than by the composition of immune cells.

### 3.5 Comparison of mutation characteristics between two groups

**Figure 5:**
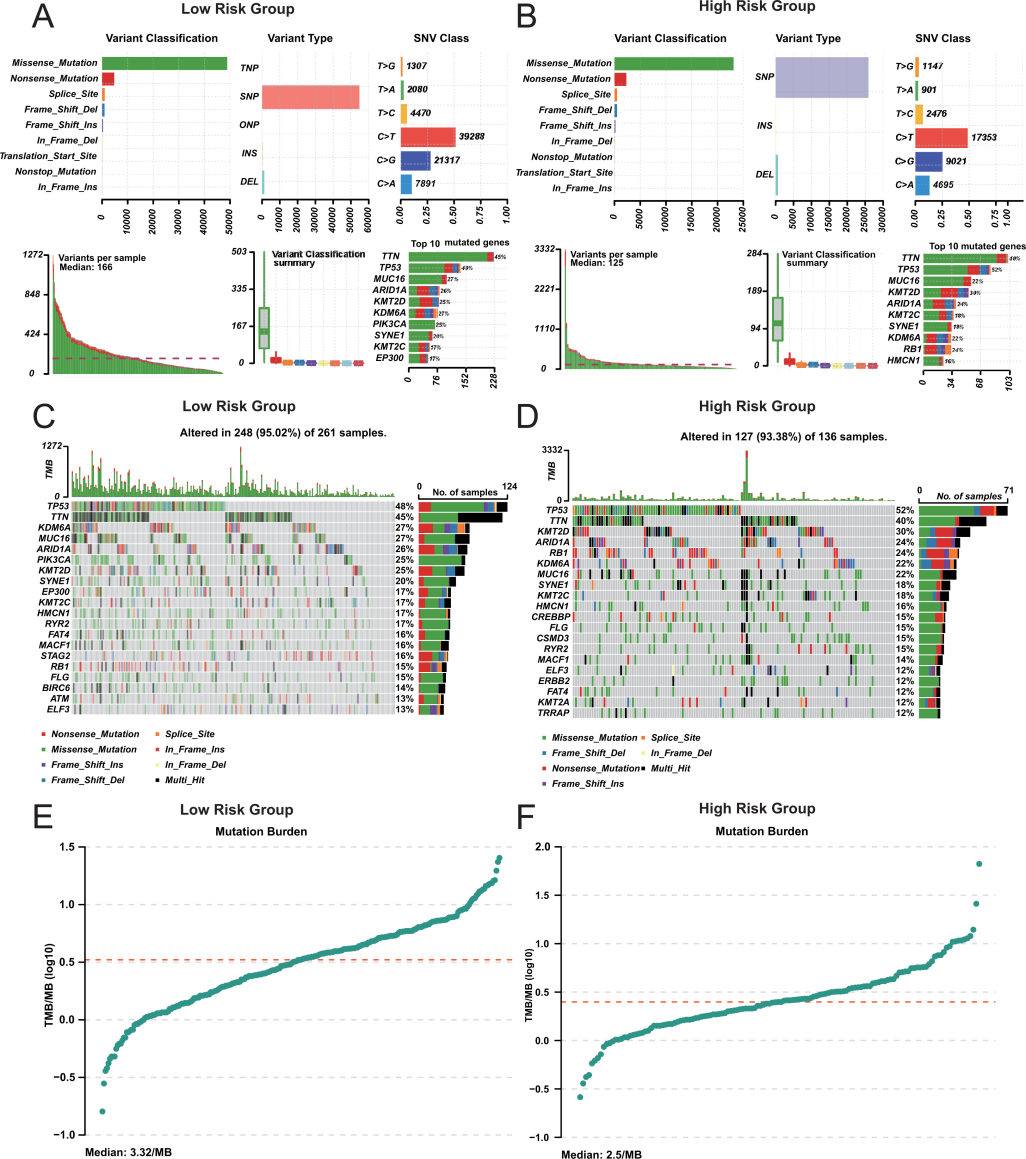
Mutation analysis in risk groups. **(A, B)** Mutation Summary in Risk Group. Variant classification and type distribution. **(C, D)** Mutation Landscape in Risk Group. **(E, F)** Tumor Mutation Burden (TMB) in Risk Group.

We analyzed the mutation profiles of the two groups to evaluate potential differences in tumor mutation burden. The number of mutations in high-risk group was higher than that in low-risk group (**Figures 5A, B**). But the mutation distribution pattern is similar between the two groups (**Figures 5C, D**). The tumor mutation burden (TMB) of high-risk group was lower than that of low-risk group (**Figures 5E-F**). Additionally, the TMB in both groups was higher than in other cancers (**Supplementary Figure S5**). Previous studies have demonstrated that patients with higher TMB tend to have prolonged survival following immune checkpoint inhibitor (ICI) therapy (37). It also confirmed the correctness of our prognosis classification.

### 3.6 Assessment of drug sensitivity in high-risk and low-risk groups

**Figure 6:**
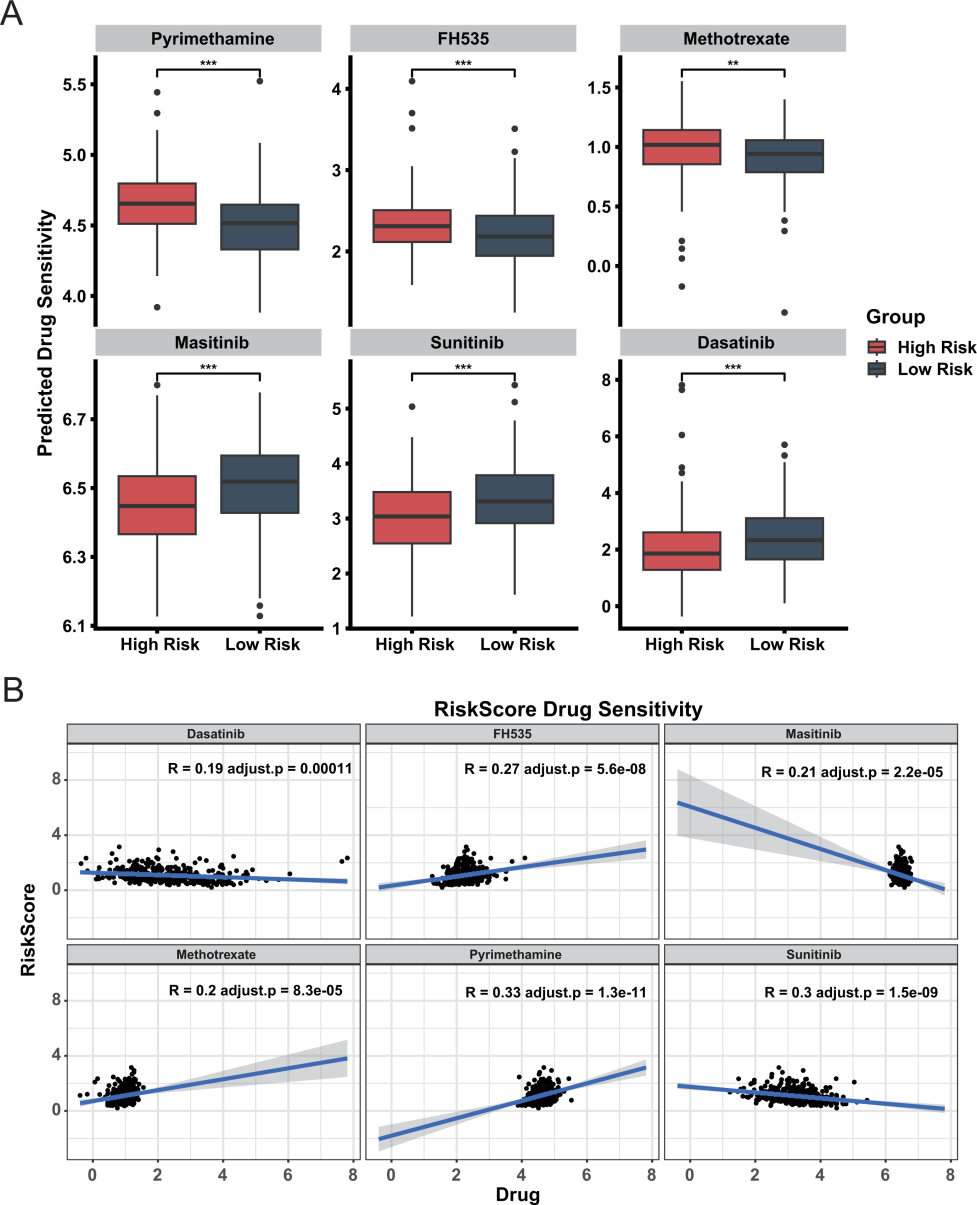
Drug sensitivity analysis in high and low risk groups. **(A)** Predicted Drug Sensitivity. Boxplots compare predicted drug sensitivity between high risk and low risk groups. Statistical significance is indicated by ** (adjust.p< 0.01) and *** (adjust.p< 0.001). **(B)** Correlation between Drug Sensitivity and Risk Score.

To guide clinical treatment for two groups, we calculated the sensitivity of each sample to various drug types, including chemotherapy agents, targeted therapies, and immune modulators. The sensitivity of high-risk group and low-risk group to drugs is different (**Figure 6A**). These findings can guide medication administration for patients in different risk groups. Additionally, a significant correlation exists between the risk score and drug sensitivity (**Figure 6B**), It shows that our CDRI model can be used to guide patients’ medication. Notably, a higher proportion of patients in the low-risk group achieved a complete treatment response compared to the high-risk group (**Supplementary Figure S6**). Recapitulation of known molecular subtypes within our prognostic groups biologically corroborates the classification logic.

### 3.7 Evaluation of the prognostic impact of CDRI combined with other scoring systems

**Figure 7:**
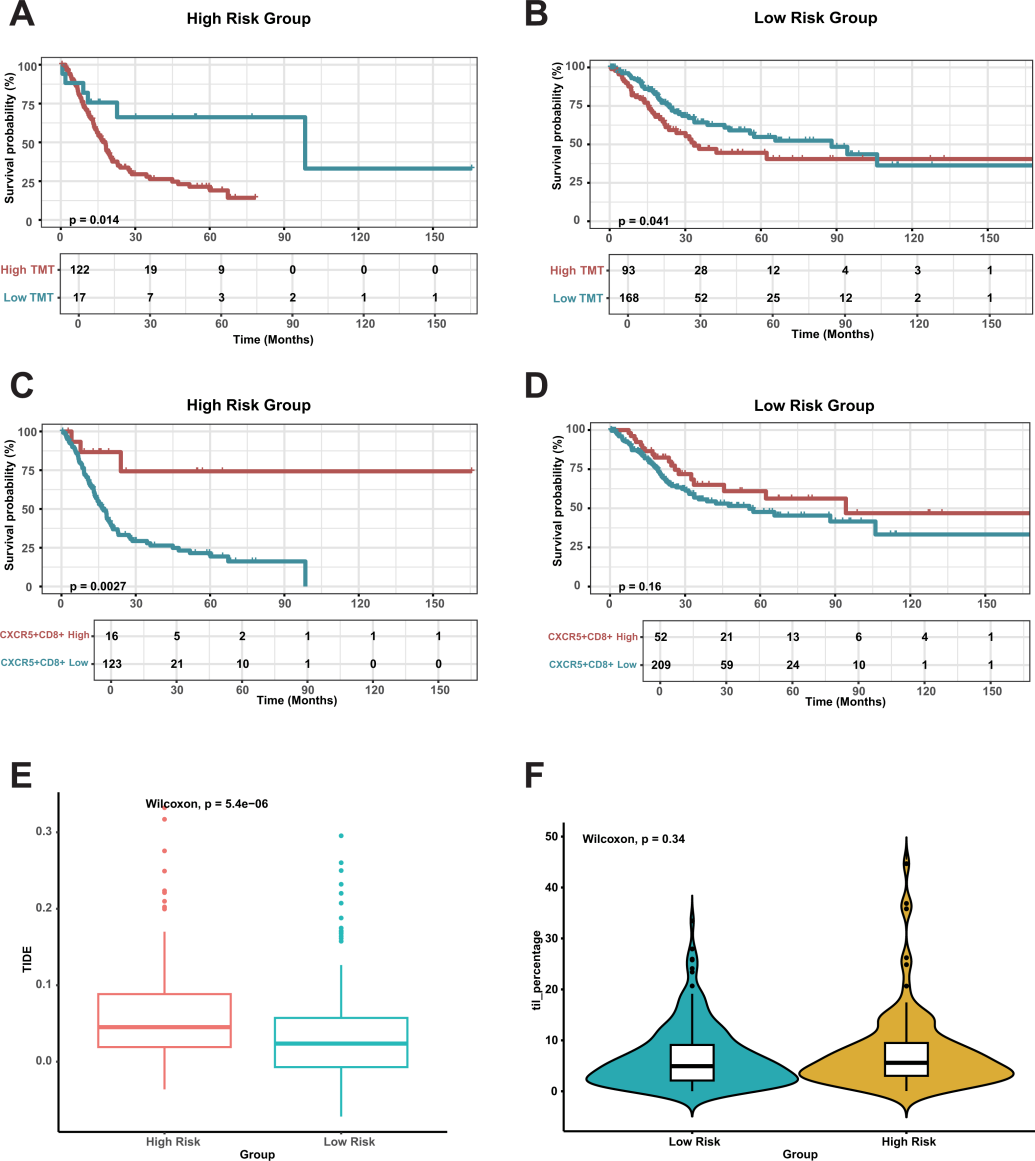
Survival analysis and immune cell impact in risk groups. **(A, B)** Overall Survival Based on Tumor Mutation Burden (TMB) in risk Group. **(C, D)** Overall Survival Based on CXCR5^+^CD8^+^ T Cells in risk Group. **(E)** Comparing TIDE scores between the risk groups. **(F)** Comparing TILs between the risk groups.

To assess the combined effect of CDRI and other evaluation scores on prognosis, we investigated the impact of TMT and CXCR5^+^CD8^+^ scores in both two groups. In the high-risk group, a higher TMT score was associated with a poorer prognosis (**Figures 7A, B**), while a higher CXCR5^+^CD8^+^ score correlated with a more favorable prognosis (**Figures 7C, D**). The TIDE score in the high-risk group was significantly higher than in the low-risk group, indicating a greater level of tumor immune escape in the former (**Figure 7E**). However, the TIDE score in both the two groups had no significant impact on patient prognosis (**Supplementary Figure S7**). Additionally, no difference in the prognosis of TIL between the two groups (**Figure 7F**; **Supplementary Figure S8**), suggesting comparable levels of tumor-infiltrating lymphocytes in both groups. This finding supports our previous conclusion that the prognostic differences between the two groups may not be solely driven by immune cell infiltration.

### 3.8 Validating the mechanism of CDRI effect based on single cell data

**Figure 8:**
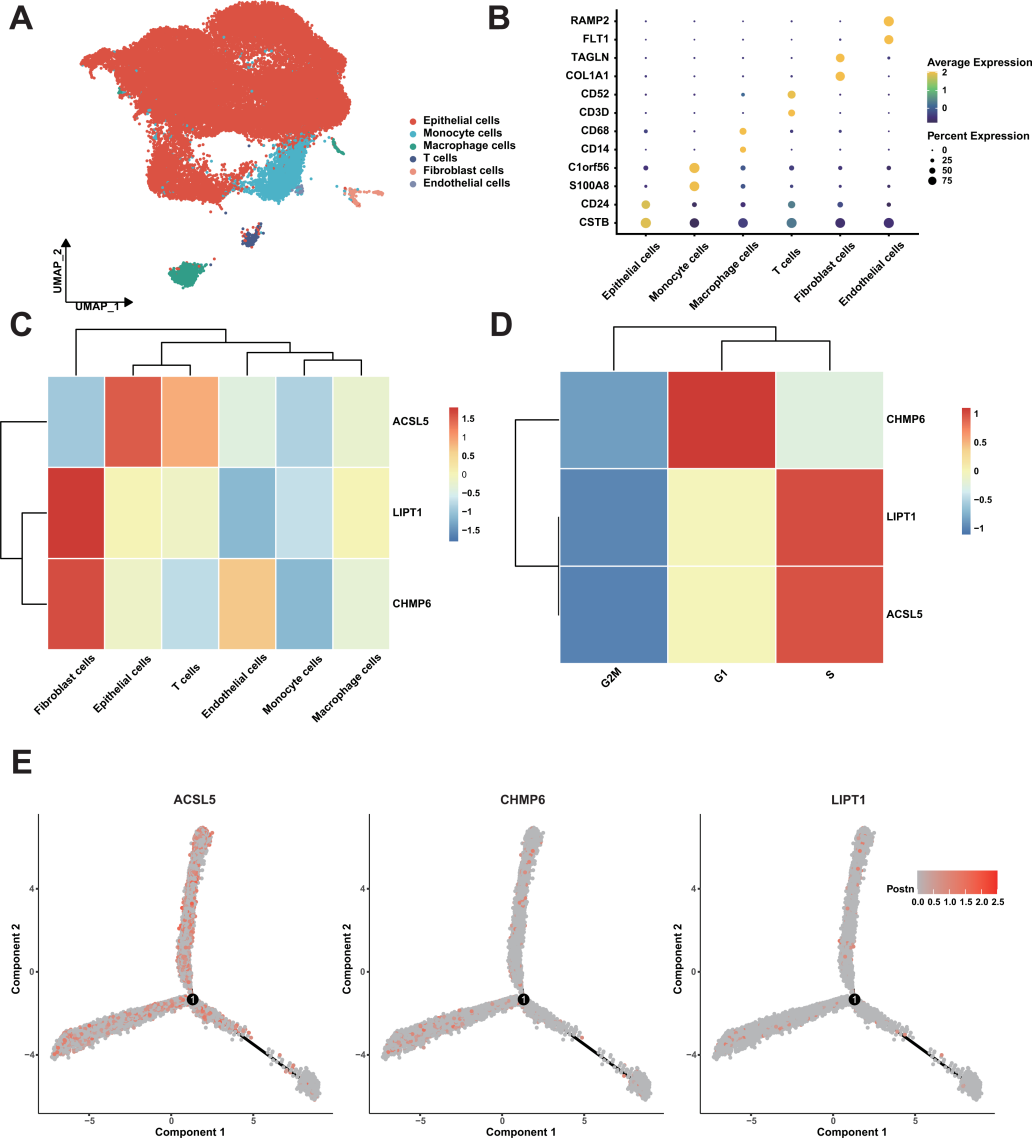
Single-Cell RNA sequencing analysis of bladder cancer samples. **(A)** UMAP Plot of cell types. **(B)** Dot plot of cell types. **(C)** Heatmap of expression of three genes in cell types. **(D)** Heatmap of expression of three genes in cell phase. **(E)** The expression distribution of three genes in the trajectory of cell differentiation.

To validate the conclusions proposed in our study at the cellular level, we conducted cluster analysis on single-cell RNA sequencing data from seven bladder cancer patients. Following cell annotation, the dataset was classified into six distinct cell types (**Figure 8A**), with epithelial cells representing the predominant population. The marker genes for each cell type were highly specific (**Figure 8B**). Notably, we observed that CHMP6 were highly expressed in fibroblasts and endothelial (**Figure 8C**), with relatively low expression in immune cells. Additionally, we found that CHMP6 expression was particularly elevated during the G1 phase, a critical phase for cell proliferation (**Figure 8D**). We also observed consistent trends in other data sets (**Supplementary Figure S9**). Further analysis indicated that the expression levels of these three genes did not significantly change during the differentiation process (**Figure 8E**). These results provide additional evidence for our hypothesis that the prognostic differences in the samples are primarily determined by tumor purity.

### 3.9 CHMP6 protein enhances BLCA cell survival and invasive migration by regulating the cell cycle

**Figure 9:**
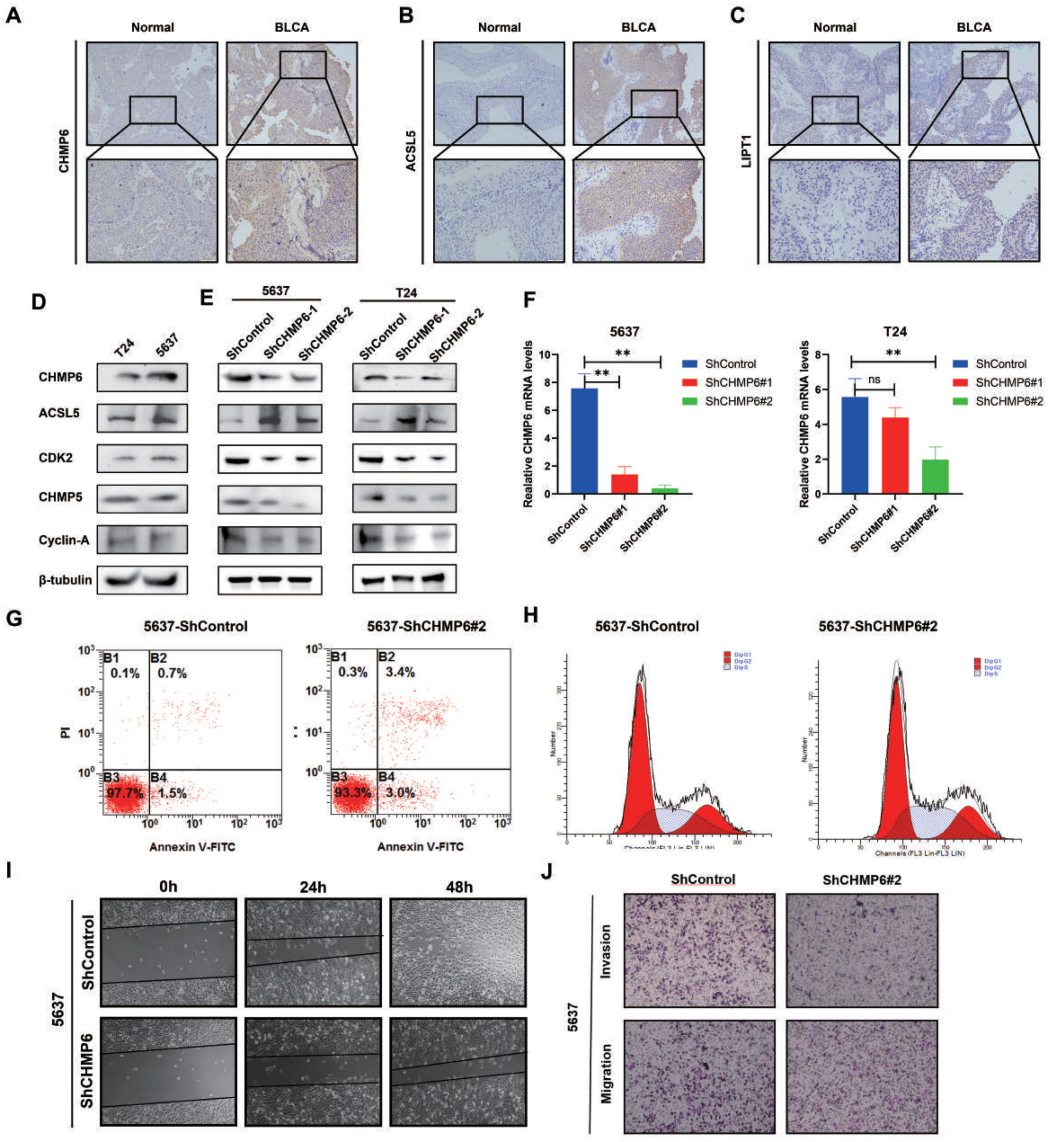
Validation of CHMP6 through *in vitro* methods. **(A-C)** Immunohistochemical Staining: Immunohistochemical analysis of CHMP6, ACSL5, and LIPT1 in BLCA tissues. **(D)** Protein Expression Levels: Expression of CHMP6 and ACSL5 proteins in 5637 and T24 cell lines. **(E)** Western Blot (WB) Analysis: Detection of CHMP6 protein knockdown in 5637 and T24 cell lines, along with ACSL5 expression. **(F)** RNA Knockdown Efficiency: Validation of CHMP6 knockdown efficiency at the RNA level in 5637 and T24 cell lines. **(G)** Apoptosis Assay: Assessment of early and late apoptosis in 5637-shControl and 5637-shCHMP6#2 cells. **(H)** Cell Cycle Analysis: Flow cytometry analysis of the cell cycle in 5637-shControl and 5637-shCHMP6#2 cells. **(I)** Migration Assay: Scratch assay to evaluate the migration ability of 5637-shControl and 5637-shCHMP6#2 cells. **(J)** Invasion and Migration Assay: Transwell assay to assess invasion and migration capabilities of 5637-shControl and 5637-shCHMP6#2 cells. ns (not significant) and ** (adjust.p < 0.01).

We investigated the cytoplasmic expression of CHMP6, ACSL5, and LIPT1 in 54 cases of BLCA using immunohistochemical analysis. In comparison to the corresponding noncancerous tissues, the protein levels of CHMP6 and ACSL5 were more highly expressed in cancerous tissues (**Figures 9A, B**) and were mainly localized in the cytoplasm and cell membranes, whereas LIPT1 was expressed at a lower level in both cancerous and paracancerous tissues (**Figure 9C**). In **Figure 9D**, we examined the expression levels of CHMP6, ACSL5, CDK2, CHMP5, Cyclin A proteins in 5637 and T24 cell lines, in which ACSL5 protein expression was lower compared to CHMP6, which was consistent with what we observed in immunohistochemistry. We constructed CHMP6 knockdown human bladder cancer cell lines and utilized WB and Q-pcr techniques for validation at the protein level and RNA level (**Figures 9D-F**), and in the cell lines with CHMP6 knockdown, we found that ACSL5 protein levels were elevated. CDK2, CHMP6 and Cyclin A protein levels were weakened (**Figure 9E**). We selected 5637-shCHMP6#2 with higher knockdown efficiency for subsequent experiments. It was found by Annexin V/PI staining that more early apoptosis and late apoptosis were exhibited in 5637-shCHMP6#2 cells (**Figure 9G**). By analyzing the cell cycle with flow cytometry, we found that 5637-shCHMP6#2 cells showed significant S-phase block (**Figure 9H**). Through the scratch assay, We observed a significant reduction in the migration ability of 5637-shCHMP6#2 cells (**Figure 9I**). In the Transwell assay, CHMP6 knockdown significantly decreased the cells’ invasive and migratory abilities (**Figure 9J**).

## 4 Discussion

The findings of this study highlight the significant prognostic role of genes involved in programmed cell death in bladder cancer. Univariate and multivariate Cox analyses further underscore the prognostic value of cell death genes. The consistency of results across the multiple datasets enhances the study’s credibility and suggests the widespread relevance of cell death-related genes across various cancer types. Immune infiltration and estimation analyses highlight the complex role of the tumor microenvironment, particularly the variations in tumor purity and stromal invasion levels. Mutation analysis suggests that a higher mutation load in the low-risk group may correlate with a more favorable prognosis. Drug sensitivity analysis may also provide practical implications for personalized treatment in clinical practice. Experimental data indicate that CHMP6 may promote the survival and invasive migration of BLCA cells by modulating the cell cycle.

Although this study offers valuable insights, several limitations must be recognized. First, the data predominantly come from public databases, such as TCGA and GEO, which may introduce sample selection bias. Furthermore, heterogeneity across datasets could affect the interpretation and generalizability of the findings. Lastly, although several prognostic genes have been identified, their underlying mechanisms and pathways warrant further investigation in future research.

Future research should consider the following directions. First, new experimental methods are needed to validate the function and mechanism of the identified genes at the single-cell level (38–40). Second, expanding the sample size, particularly to include individuals from diverse racial and geographical backgrounds, would help assess the generalizability of the findings. Exploring the transcriptional mechanism of chmp6 in paracancerous tissues and tumors will provide direction for the study of bladder cancer treatment. Additionally, integrating various data sources and analytical techniques could strengthen the reliability and broader applicability of the results. Finally, the drug sensitivity analysis in this study may serve as a valuable resource for designing clinical trials of relevant drugs, aiding in the evaluation of their potential for personalized bladder cancer treatment.

## 5 Conclusions

In this study, we developed a comprehensive death gene risk model (CDRI) based on cell death-related genes. Additionally, Our analysis indicates that the prognosis in cancer samples is mainly related to tumor purity. The CHMP6 protein promotes BLCA cell survival and invasive migration through modulation of the cell cycle. Our findings deepen the molecular understanding of bladder cancer and offer potential biomarkers and therapeutic targets to improve patient prognosis.

## Data Availability

The data presented in the study are deposited in the GEO repository, accession number GSE32548, GSE32894, GSE31684, GSE129845, GSE277524, GSE135337. The data presented in the study are deposited in the TCGA repository, accession number TCGA-BLCA, TCGA-UCEC, TCGA-LIHC, TCGA-MESO, TCGA-ACC.

## Supplementary material

Supplementary Figure S1 Analysis of Differential Gene Expression and Specific Gene Expression Levels. **(A)** Intersection of Differentially Expressed Genes and Copper-Related Genes. Bar plot shows the intersection sizes of upregulated and downregulated genes with copper-related genes. The set size indicates the total number of genes in each category. **(B)** Intersection of Differentially Expressed Genes and Ferroptosis-Related Genes. **(C)** Intersection of Differentially Expressed Genes and Immunogenic Cell Death (ICD)-Related Genes. **(D)** ACSL5 Expression in Normal and Tumor Samples. Box plot shows the expression levels of ACSL5 in normal and tumor samples. Expression is measured in log2(TPM + 1). **(E)** CHMP6 Expression in Normal and Tumor Samples. **(F)** LIPT1 Expression in Normal and Tumor Samples.

Supplementary Figure S2 Performance of CDRI model in independent validation dataset. **(A)** The performance of CDRI model in GSE32548 dataset, the left figure is the survival KM curve, and the right figure is the ROC of CDRI model. **(B)** The performance of CDRI model in GSE32894 dataset, the left figure is the survival KM curve, and the right figure is the ROC of CDRI model.

Supplementary Figure S3 ROC of CDRI model in multiple datasets.

Supplementary Figure S4 Comparison of immune cell proportion estimated by various methods. **(A)** Comparison of immune cell proportion in different risk groups estimated by xCell method. **(B)** Comparison of immune cell proportion in different risk groups estimated by TIMER method. **(C)** Comparison of immune cell proportion in different risk groups estimated by EPIC method. **(D)** Comparison of immune cell proportion in different risk groups estimated by QUANTISEQ method. **(E)** Comparison of different active states of T cells.

Supplementary Figure S5 Tumor Mutation Burden Across Various Cancer Types.

Supplementary Figure S6 Treatment Response Comparison Between High and Low Risk Groups.

Supplementary Figure S7 Survival Analysis Based on Tumor Immune Dysfunction and Exclusion (TIDE) Score.

Supplementary Figure S8 Comparison of Immune Infiltration Between High and Low Risk Groups.

Supplementary Figure S9 Specific enrichment of genes in cell type and cell cycle. The left figure shows the expression of three genes in different cell types, and the right figure shows the expression of three genes in different cell cycles.

Supplementary Table S1 Three kinds of cell death related genes.

Supplementary Table S2 Different results of different groups.

Supplementary Table S3 Results of univariate Cox analysis.

Supplementary Table S4 Grouping results of samples.

Supplementary Table S5 Baseline characteristics of different group patient.

Supplementary Table S6 C_index of CDRI model on different datasets.
