# Supplementary figures and images for: CHMP6 as a novel prognostic biomarker in bladder cancer: insights from a comprehensive cell death-related gene risk model
Source: Front Oncol. 2025 Jun 24;15:1564826. doi: 10.3389/fonc.2025.1564826 (PMC12234533; doi:10.3389/fonc.2025.1564826)

# Supplement Figure 3

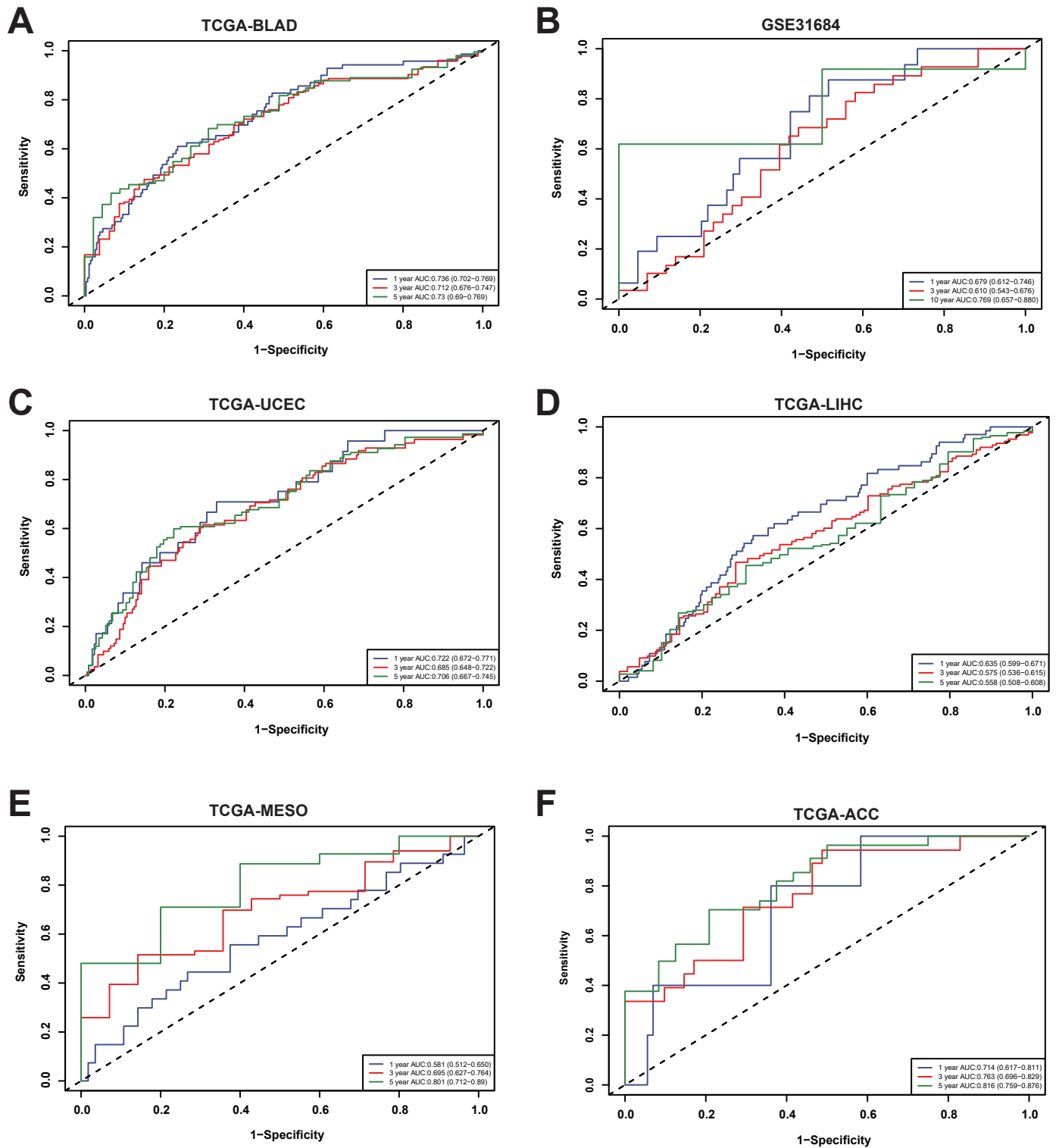

Supplement: Supplementary Figure S1 — Analysis of Differential Gene Expression and Specific Gene Expression Levels. (A) Intersection of Differentially Expressed Genes and Copper-Related Genes. Bar plot shows the intersection sizes of upregulated and downregulated genes with copper-related genes. The set size indicates the total number of genes in each category. (B) Intersection of Differentially Expressed Genes and Ferroptosis-Related Genes. (C) Intersection of Differentially Expressed Genes and Immunogenic Cell Death (ICD)-Related Genes. (D) ACSL5 Expression in Normal and Tumor Samples. Box plot shows the expression levels of ACSL5 in normal and tumor samples. Expression is measured in log2(TPM + 1). (E) CHMP6 Expression in Normal and Tumor Samples. (F) LIPT1 Expression in Normal and Tumor Samples. [file DataSheet1.zip › supplementfigure3.pdf]

# Supplement Figure 4

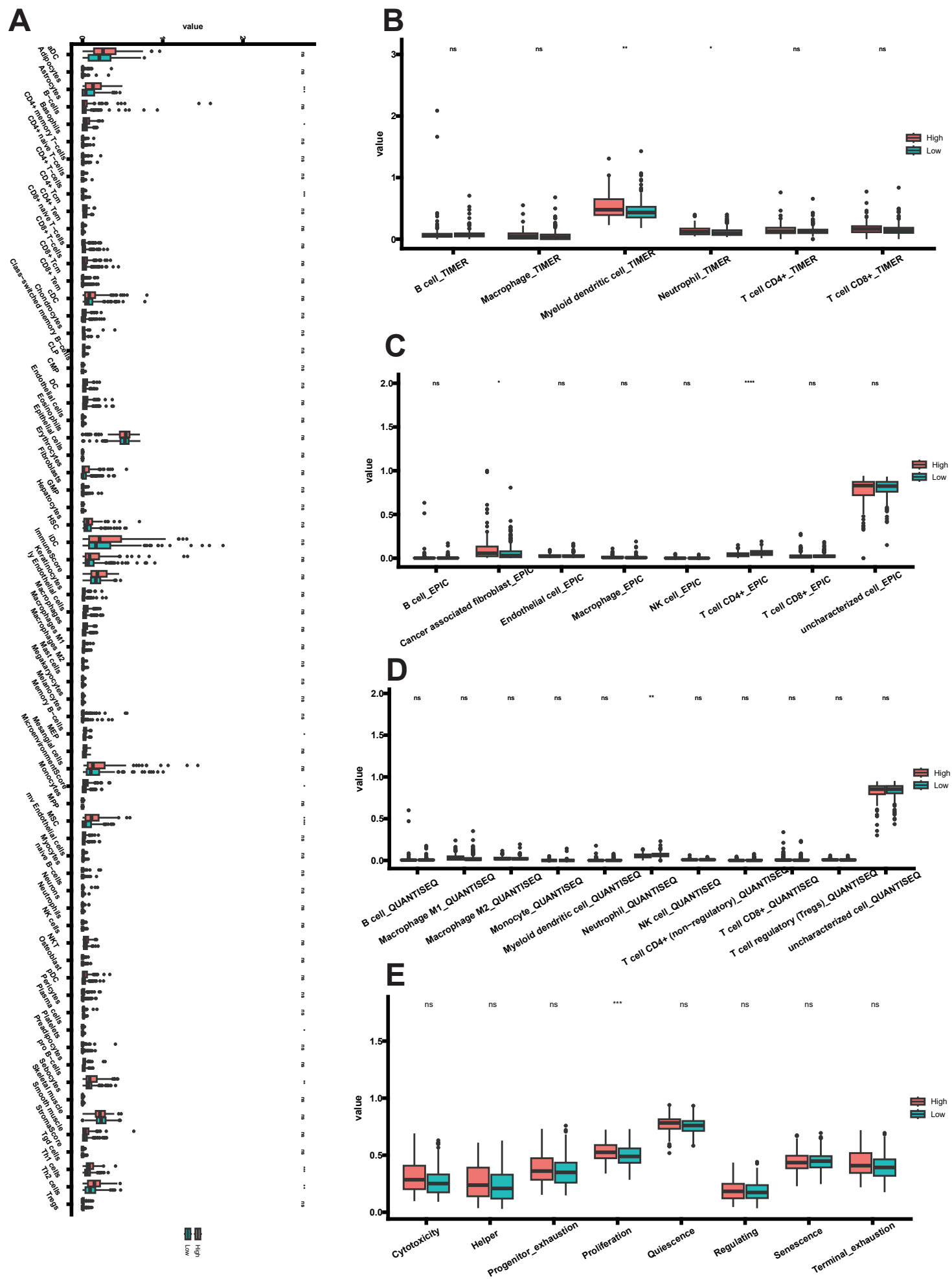

Supplement: Supplementary Figure S1 — Analysis of Differential Gene Expression and Specific Gene Expression Levels. (A) Intersection of Differentially Expressed Genes and Copper-Related Genes. Bar plot shows the intersection sizes of upregulated and downregulated genes with copper-related genes. The set size indicates the total number of genes in each category. (B) Intersection of Differentially Expressed Genes and Ferroptosis-Related Genes. (C) Intersection of Differentially Expressed Genes and Immunogenic Cell Death (ICD)-Related Genes. (D) ACSL5 Expression in Normal and Tumor Samples. Box plot shows the expression levels of ACSL5 in normal and tumor samples. Expression is measured in log2(TPM + 1). (E) CHMP6 Expression in Normal and Tumor Samples. (F) LIPT1 Expression in Normal and Tumor Samples. [file DataSheet1.zip › supplementfigure4.pdf]

# Supplement Figure 5

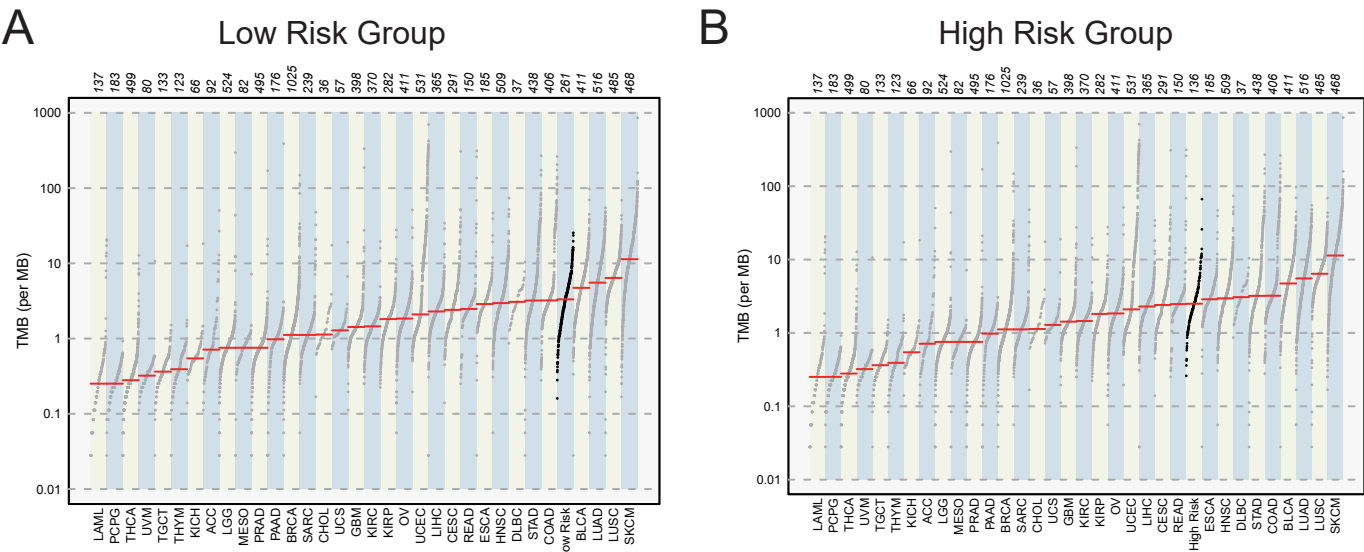

Supplement: Supplementary Figure S1 — Analysis of Differential Gene Expression and Specific Gene Expression Levels. (A) Intersection of Differentially Expressed Genes and Copper-Related Genes. Bar plot shows the intersection sizes of upregulated and downregulated genes with copper-related genes. The set size indicates the total number of genes in each category. (B) Intersection of Differentially Expressed Genes and Ferroptosis-Related Genes. (C) Intersection of Differentially Expressed Genes and Immunogenic Cell Death (ICD)-Related Genes. (D) ACSL5 Expression in Normal and Tumor Samples. Box plot shows the expression levels of ACSL5 in normal and tumor samples. Expression is measured in log2(TPM + 1). (E) CHMP6 Expression in Normal and Tumor Samples. (F) LIPT1 Expression in Normal and Tumor Samples. [file DataSheet1.zip › supplementfigure5.pdf]

# Supplement Figure 6

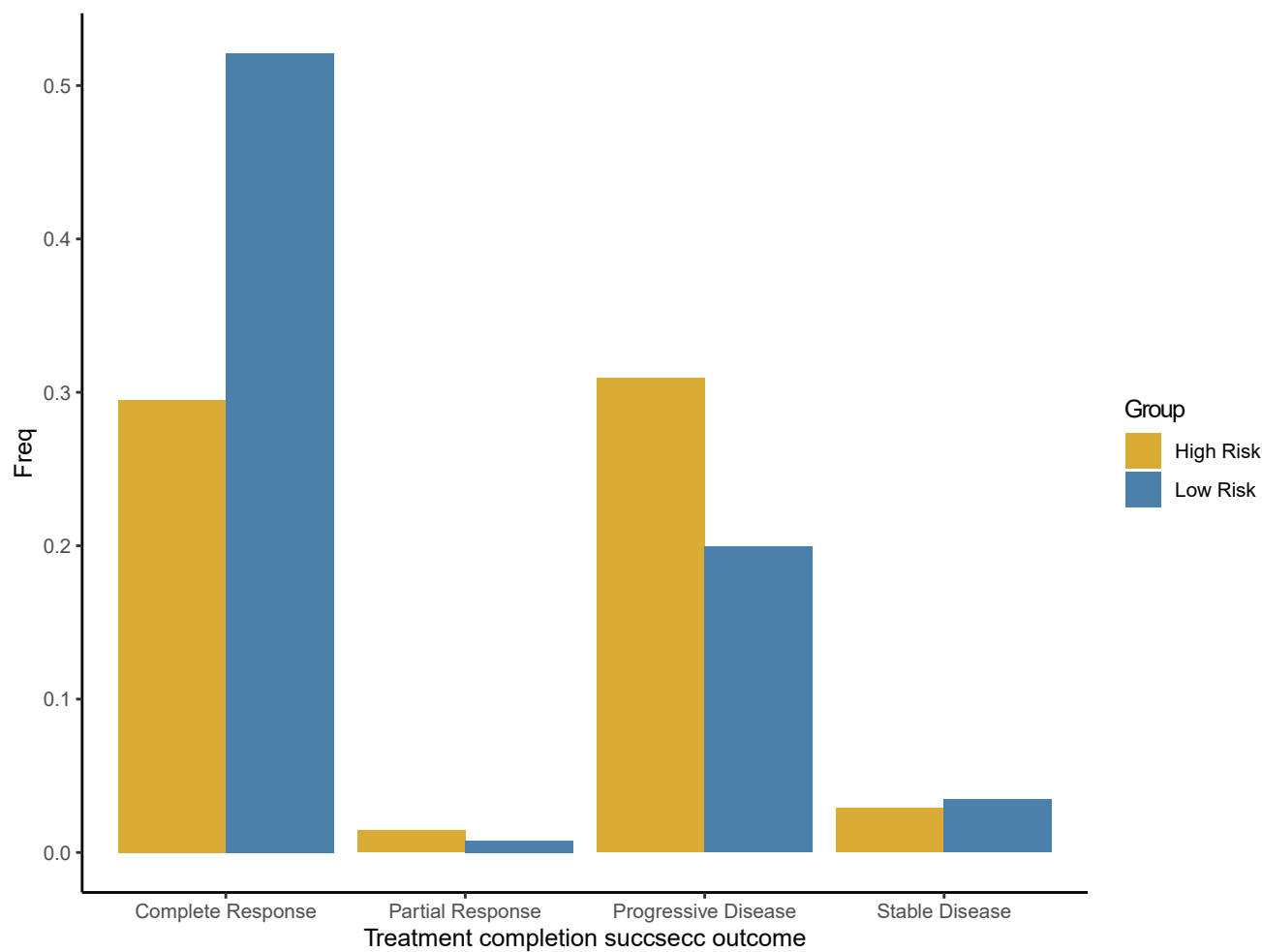

Supplement: Supplementary Figure S1 — Analysis of Differential Gene Expression and Specific Gene Expression Levels. (A) Intersection of Differentially Expressed Genes and Copper-Related Genes. Bar plot shows the intersection sizes of upregulated and downregulated genes with copper-related genes. The set size indicates the total number of genes in each category. (B) Intersection of Differentially Expressed Genes and Ferroptosis-Related Genes. (C) Intersection of Differentially Expressed Genes and Immunogenic Cell Death (ICD)-Related Genes. (D) ACSL5 Expression in Normal and Tumor Samples. Box plot shows the expression levels of ACSL5 in normal and tumor samples. Expression is measured in log2(TPM + 1). (E) CHMP6 Expression in Normal and Tumor Samples. (F) LIPT1 Expression in Normal and Tumor Samples. [file DataSheet1.zip › Supplementfigure6.pdf]

# Supplement Figure 7

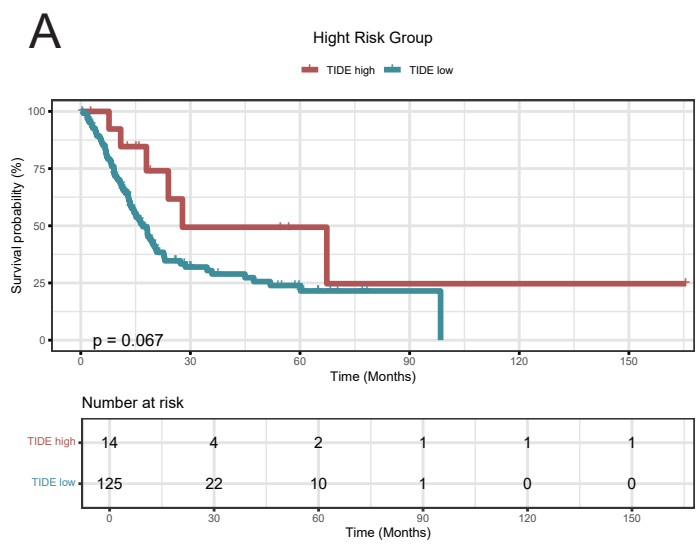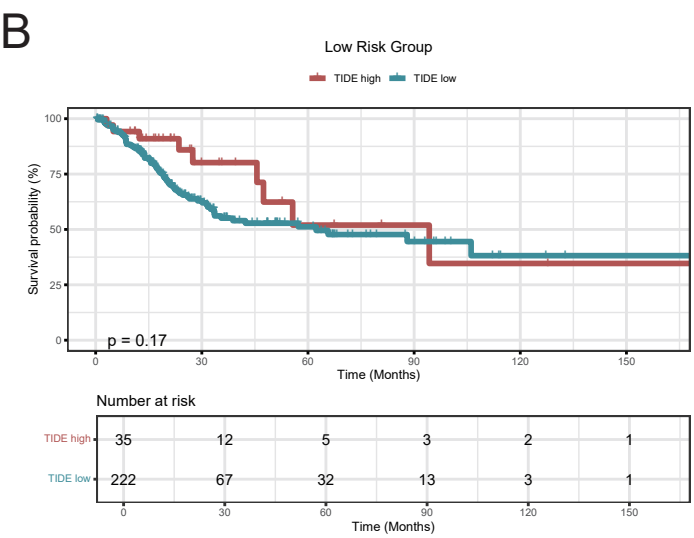

Supplement: Supplementary Figure S1 — Analysis of Differential Gene Expression and Specific Gene Expression Levels. (A) Intersection of Differentially Expressed Genes and Copper-Related Genes. Bar plot shows the intersection sizes of upregulated and downregulated genes with copper-related genes. The set size indicates the total number of genes in each category. (B) Intersection of Differentially Expressed Genes and Ferroptosis-Related Genes. (C) Intersection of Differentially Expressed Genes and Immunogenic Cell Death (ICD)-Related Genes. (D) ACSL5 Expression in Normal and Tumor Samples. Box plot shows the expression levels of ACSL5 in normal and tumor samples. Expression is measured in log2(TPM + 1). (E) CHMP6 Expression in Normal and Tumor Samples. (F) LIPT1 Expression in Normal and Tumor Samples. [file DataSheet1.zip › supplementfigure7.pdf]

# Supplement Figure 8

A

High Risk group

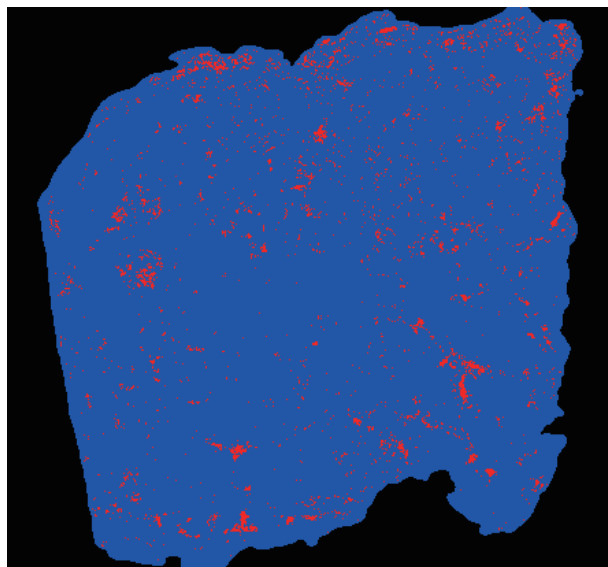

B

Low Risk group

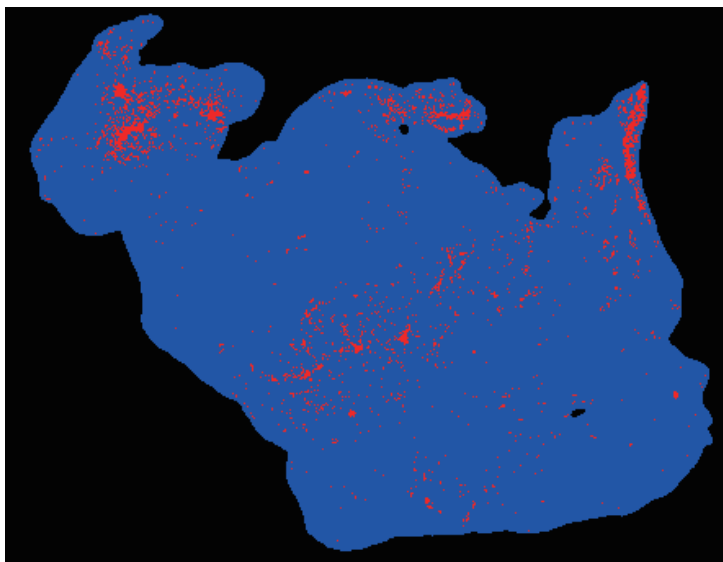

Supplement: Supplementary Figure S1 — Analysis of Differential Gene Expression and Specific Gene Expression Levels. (A) Intersection of Differentially Expressed Genes and Copper-Related Genes. Bar plot shows the intersection sizes of upregulated and downregulated genes with copper-related genes. The set size indicates the total number of genes in each category. (B) Intersection of Differentially Expressed Genes and Ferroptosis-Related Genes. (C) Intersection of Differentially Expressed Genes and Immunogenic Cell Death (ICD)-Related Genes. (D) ACSL5 Expression in Normal and Tumor Samples. Box plot shows the expression levels of ACSL5 in normal and tumor samples. Expression is measured in log2(TPM + 1). (E) CHMP6 Expression in Normal and Tumor Samples. (F) LIPT1 Expression in Normal and Tumor Samples. [file DataSheet1.zip › supplementfigure8.pdf]

# Supplement Figure 9

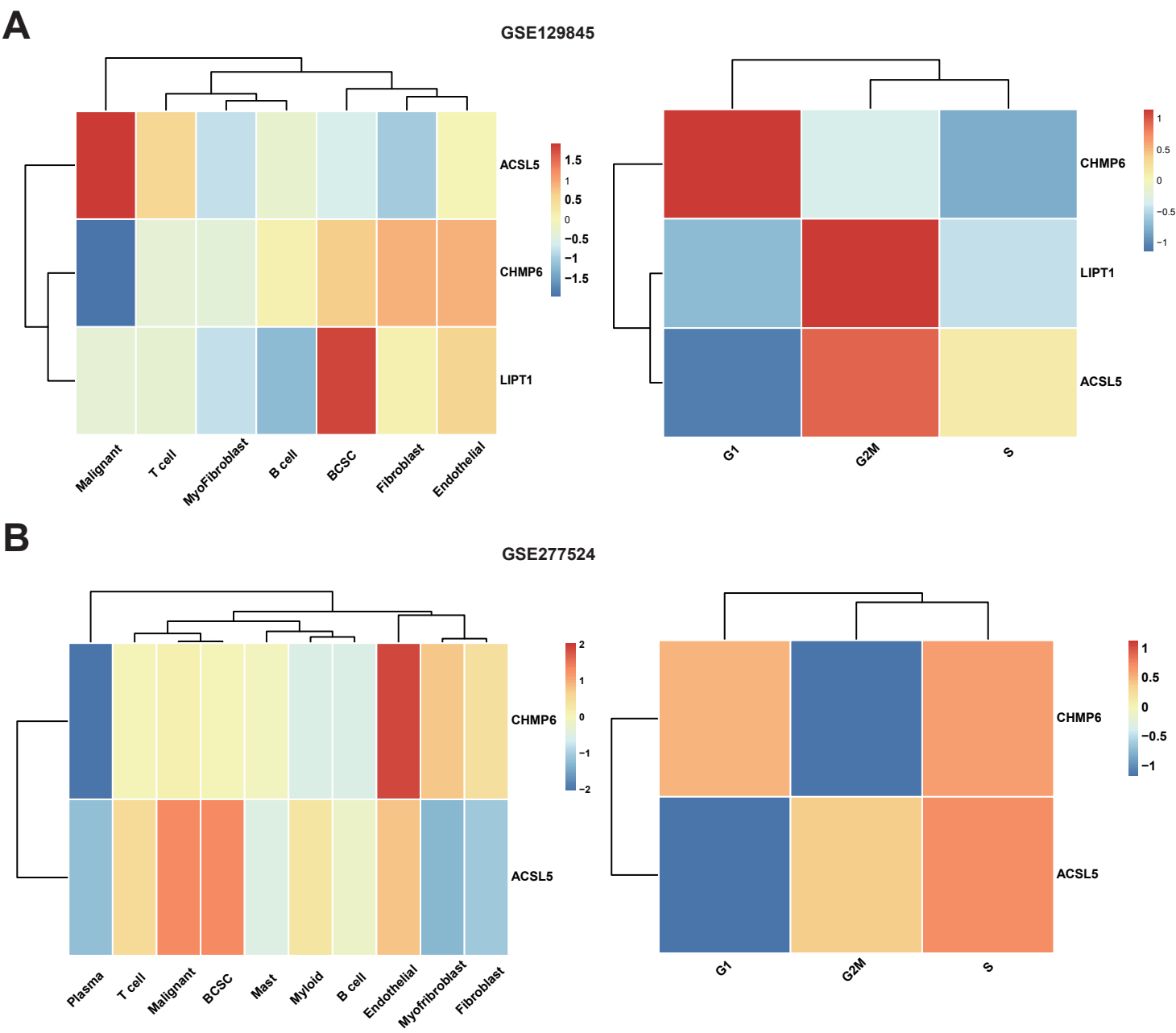

Supplement: Supplementary Figure S1 — Analysis of Differential Gene Expression and Specific Gene Expression Levels. (A) Intersection of Differentially Expressed Genes and Copper-Related Genes. Bar plot shows the intersection sizes of upregulated and downregulated genes with copper-related genes. The set size indicates the total number of genes in each category. (B) Intersection of Differentially Expressed Genes and Ferroptosis-Related Genes. (C) Intersection of Differentially Expressed Genes and Immunogenic Cell Death (ICD)-Related Genes. (D) ACSL5 Expression in Normal and Tumor Samples. Box plot shows the expression levels of ACSL5 in normal and tumor samples. Expression is measured in log2(TPM + 1). (E) CHMP6 Expression in Normal and Tumor Samples. (F) LIPT1 Expression in Normal and Tumor Samples. [file DataSheet1.zip › supplementfigure9.pdf]

# Supplement Figure 1

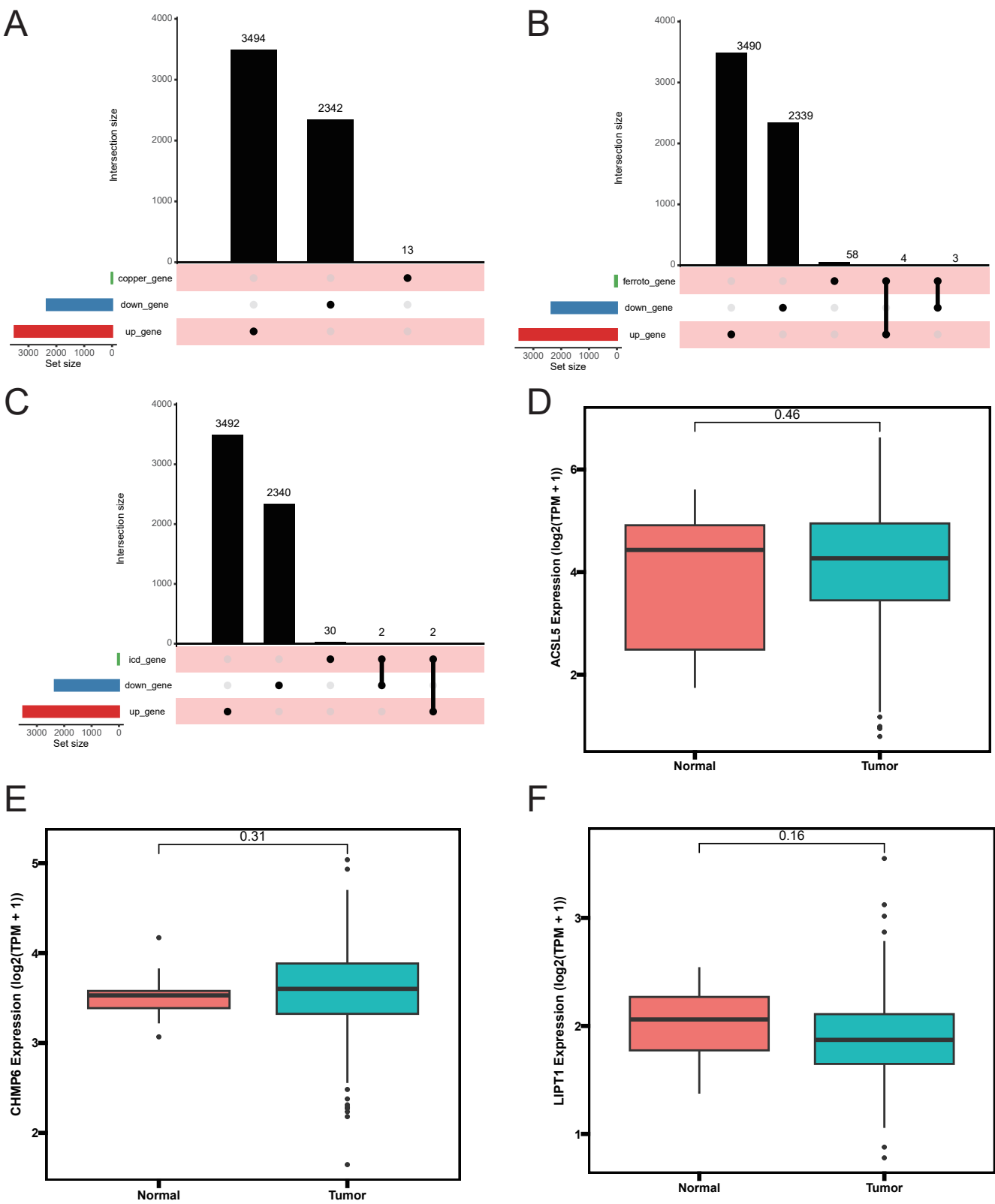

Supplement: Supplementary Figure S1 — Analysis of Differential Gene Expression and Specific Gene Expression Levels. (A) Intersection of Differentially Expressed Genes and Copper-Related Genes. Bar plot shows the intersection sizes of upregulated and downregulated genes with copper-related genes. The set size indicates the total number of genes in each category. (B) Intersection of Differentially Expressed Genes and Ferroptosis-Related Genes. (C) Intersection of Differentially Expressed Genes and Immunogenic Cell Death (ICD)-Related Genes. (D) ACSL5 Expression in Normal and Tumor Samples. Box plot shows the expression levels of ACSL5 in normal and tumor samples. Expression is measured in log2(TPM + 1). (E) CHMP6 Expression in Normal and Tumor Samples. (F) LIPT1 Expression in Normal and Tumor Samples. [file DataSheet1.zip › supplementfigure1.pdf]

# Supplement Figure 2

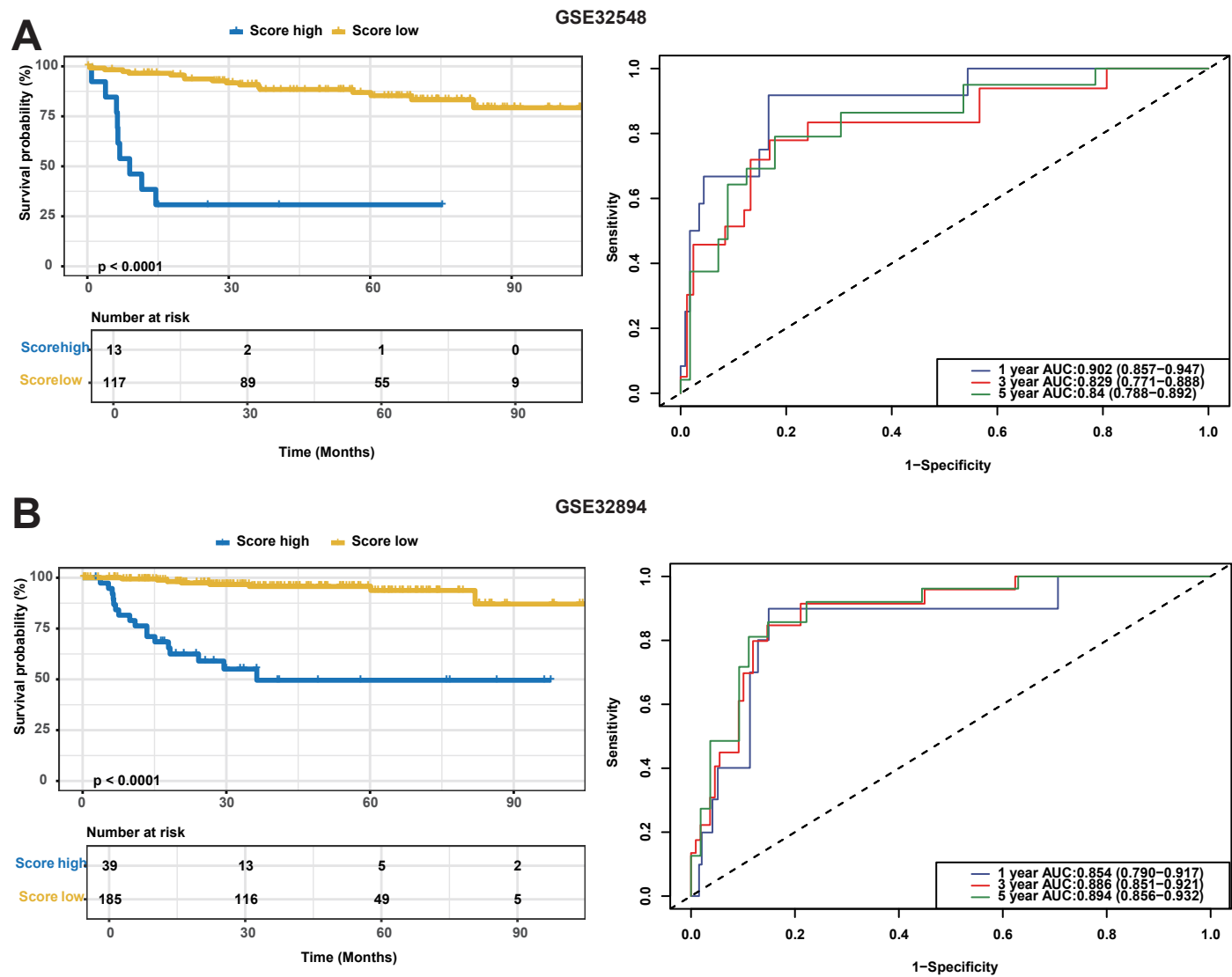

Supplement: Supplementary Figure S1 — Analysis of Differential Gene Expression and Specific Gene Expression Levels. (A) Intersection of Differentially Expressed Genes and Copper-Related Genes. Bar plot shows the intersection sizes of upregulated and downregulated genes with copper-related genes. The set size indicates the total number of genes in each category. (B) Intersection of Differentially Expressed Genes and Ferroptosis-Related Genes. (C) Intersection of Differentially Expressed Genes and Immunogenic Cell Death (ICD)-Related Genes. (D) ACSL5 Expression in Normal and Tumor Samples. Box plot shows the expression levels of ACSL5 in normal and tumor samples. Expression is measured in log2(TPM + 1). (E) CHMP6 Expression in Normal and Tumor Samples. (F) LIPT1 Expression in Normal and Tumor Samples. [file DataSheet1.zip › supplementfigure2.pdf]
